# Oral Health Disparities and Unmet Dental Needs among Preschool Children in Chelsea, MA: Exploring Mechanisms, Defining Solutions

**DOI:** 10.4172/2332-0702.1000138

**Published:** 2014-06-01

**Authors:** Inyang Isong, Laila Dantas, Macda Gerard, Karen Kuhlthau

**Affiliations:** 1 Boston Children’s Hospital, Boston, MA, USA; 2 Harvard Medical School, Boston, MA, USA; 3 Cambridge Health Alliance, Cambridge, MA, USA; 4 Harvard School of Dental Medicine, Boston, MA, USA; 5 Department of Public Health, Brown University, Providence, RI, USA; 6 Center for Child & Adolescent Health Research and Policy, MGHfC, Boston, MA, USA

**Keywords:** Children, Preventive dental care, Oral health, Early childhood caries

## Abstract

**Background:**

Significant disparities exist in children’s receipt of preventive dental care (PDC) in the United States. Many of the children at greatest risk of dental disease do not receive timely PDC; when they do receive dental care, it is often more for relief of dental pain. Chelsea is a low-income, diverse Massachusetts community with high rates of untreated childhood caries. There are various dental resources available in Chelsea, yet many children do not access dental care at levels equivalent to their needs.

**Objective:**

Using Chelsea as a case-study, to explore factors contributing to forgone PDC (including the age 1 dental visit) in an in-depth way.

**Methods:**

We used a qualitative study design that included semi-structured interviews with parents of preschool children residing in Chelsea, and Chelsea-based providers including pediatricians, dentists, a dental hygienist and early childhood care providers. We examined: a) parents’ dental attitudes and oral health cultural beliefs; b) parents’ and providers’ perspectives on facilitators and barriers to PDC, reasons for unmet needs, and proposed solutions to address the problem. We recorded, transcribed and independently coded all interviews. Using rigorous, iterative qualitative data analyses procedures, we identified emergent themes.

**Results:**

Factors perceived to facilitate receipt of PDC included Head-Start oral health policies, strong pediatric primary care/dental linkages, community outreach and advertising, and parents’ own oral health experiences. Most parents and providers perceived there to be an adequate number of accessible dental services and resources in Chelsea, including for Medicaid enrollees. However, several barriers impeded children from receiving timely PDC, the most frequently cited being insurance related problems for children and adults. Other barriers included limited dental services for children <2 years, perceived poor quality of some dental practices, lack of emphasis on prevention-based dental care, poor care-coordination, and insufficient culturally-appropriate care. Important family-level barriers included parental oral health literacy, cultural factors, limited English proficiency and competing priorities. Several solutions were proposed to address identified barriers.

**Conclusion:**

Even in a community with a considerable number of dental resources, various factors may preclude access to these services by preschool-aged children. Opportunities exist to address modifiable factors through strategic oral health policies, community outreach and improved care coordination between physicians, dentists and early childhood care providers.

## Introduction

Dental care is the greatest unmet health care need among children in the United States (US) [1]. Through regular preventive dental care (PDC), many oral problems can be prevented or treated early, thereby averting complications. However, children at greatest risk of dental disease and its complications tend not to receive timely PDC; when they do receive care; it is often more for treatment of symptoms [2]. Disparities in receipt of PDC exist among minority and low-income children, who on average have fewer dental visits and poorer oral health (OH) status than their white or higher-income peers [3–5]. Nationally, Hispanic children have the lowest rates of dental care utilization [6]. Racial minority children, particularly Native-American and Hispanic children also suffer a disproportionate burden of oral disease [2,7–10]. They have higher rates of untreated caries, greater disease severity and are more likely to experience complications of untreated caries [7].

Preschool-aged children (ages 1–5 years) are considered another vulnerable population at risk of poor OH, and have some of the lowest rates of PDC utilization among US children. According to the National Survey of Children’s Health, 45.7% of US children aged ≤ 5 years have never had a preventive dental visit, compared to rates of 12.4% and 14.9% among children aged 6–11 years and 12–17 years, respectively [11]. Preventive dental care should occur periodically, (typically every 6 months) and comprises counseling on how to optimize OH (e.g. dietary education, dental trauma prevention, oral hygiene) and delivery of caries prevention strategies [12–14] like fluoride and sealant applications [15]. The American Academy of Pediatrics and American Academy of Pediatric Dentists recommend that children at risk of developing dental caries should be directed to establish a dental home 6 months after eruption of the first tooth, and no later than 12 months of age [16–17]. Early establishment of preventive dental care can help optimize children’s OH through prevention, early detection and management of oral conditions. A study by Biel et al. [18] found that receipt of visits early in life among a population of Medicaid-enrolled children in North Carolina helped to suppress disease among high risk children. However, these children were more likely to seek care on an urgent basis than for preventive care. That study underscored the need to evaluate ways to enhance dental care access for young children enrolled in Medicaid.

Several studies have identified factors that contribute to low use of dental services by minority and low income children, some of which include insurance status, parental education, low reimbursement rates and a geographic mal-distribution of dentists [3,19]. However, most of these studies have focused on older, school-aged children; the literature is limited on multi-level contextual factors that may contribute to PDC utilization among preschool-aged children, particularly the age 1 year dental visit. A recent study identified barriers that dentists perceived precluded them from providing care to preschool-aged children [20]. These included poor reimbursement levels, low comfort level treating young children, and need for additional training. The goal of our study was to examine facilitators and barriers to receipt of PDC among preschool-aged children residing in a low-income, predominantly minority community in Massachusetts. We sought to explore multilevel, contextual factors that may contribute to unmet dental needs or lack of receipt of PDC (particularly the age 1 year dental visit) among a vulnerable population of preschool-aged children residing in Chelsea, MA. Our findings could shed light on potential strategies to enhance dental care access and utilization for young children.

## Materials and Methods

### Study setting

The study was conducted in Chelsea-a densely populated community in Massachusetts, with a land area of 1.8 square miles [21]. In the community reside a large number of Hispanic, recent immigrant and African refugee families. Chelsea is unique in terms of availability of OH services and programs. According to the MassHealth (MA Medicaid) website, there are currently about 12 dental practices that accept MassHealth within a 1 mile square radius from the Chelsea city center, and provide care for preschool-aged children <5 years [22]. Nine of these practices reportedly see children as young as 1 year of age. Additional OH community resources include free dental services and programs delivered through mobile dental vans, daycare, Head-Start, schools and other community locations. Children can also receive free, comprehensive dental care in a Chelsea school-based program. In spite of these services, significant OH problems exist in the community; about 38% of school-aged children have untreated caries-significantly higher than the national average [23]. Further, many children do not receive timely PDC, and available services are often underutilized. It is not clear why even within this community with residents that face similar barriers, children have disparate patterns of dental utilization; some children are more likely than their equally disadvantaged peers in Chelsea to forego PDC. This could be as a result of additional contributory factors such as personal or structural barriers, community OH attitudes, cultural beliefs and acculturation that have not been well studied. There is a need to assess underlying mechanisms that foster these differences, as it could inform development of community based interventions that target identified barriers. We therefore chose Chelsea as a case study to explore in an in-depth way, contextual factors at play in an urban low-income, racially diverse community and obtain community input on solutions to address them.

### Study design

**Figure 1:**
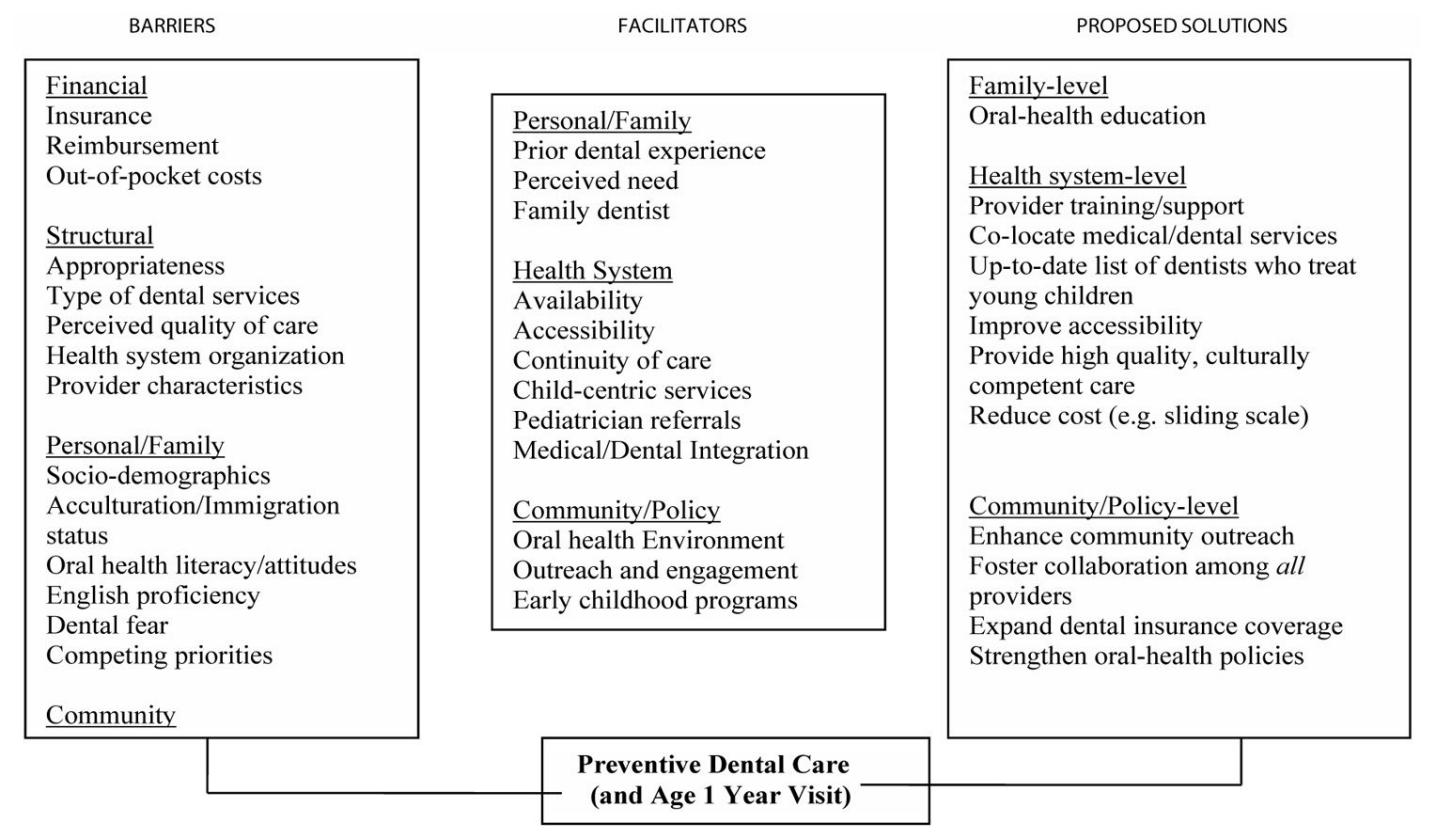
Conceptual Framework of Influences on Preschool-children’s Receipt of Preventive Dental Care.

We used a qualitative study design that comprised: 1) semi-structured interviews with parents of preschool-aged children residing in Chelsea, MA; 2) brief parent surveys that elicited parent and child socio-demographics, children’s dental visit history and parent knowledge of community dental resources; and 3) key informant interviews with providers, including clinicians (pediatricians, dentists, hygienist) and early childhood program providers, who provided multiple perspectives on the problem. We aimed to assess in an indepth way: a) parents’ and providers’ views on facilitators and barriers to PDC and reasons for unmet needs; b) parents’ dental attitudes and OH cultural beliefs, and how these influence the age 1 year dental visit and receipt of PDC in general; c) parents’ and providers’ proposed solutions to address the problem. The study was guided by our conceptual framework (Figure 1), which we developed using constructs drawn from some pertinent conceptual models that have been used to study various factors that influence children’s OH and dental utilization [24,25].

### Study participants and recruitment strategy

Parents of all children aged 1–5 years that resided in Chelsea during the study period were eligible for parent interviews. So as to get a diverse sample, we recruited parents of eligible children from three diverse sites: Massachusetts General Hospital Chelsea Community Health Center (MCCHC) Pediatric Clinic; the Intergenerational Literacy Program, (a Chelsea-based program that offers literacy instruction to parents and supports families in working with their children); and the Healthy Families/Young Parent Program at Roca (a youth, family, and community development organization located in Chelsea). We selected participants from MCCHC by identifying eligible children who had a medical visit at the clinic within the previous 12 months. For recruitment, we used purposive, stratified sampling techniques, based on the following criteria-based categories: child’s race/ethnicity, socio-economic status, age, and history of preventive dental visit. We mailed invitational letters describing the study to a randomized sample of parents of children drawn from each criteria-defined group and followed-up with phone calls to enroll parents who did return enclosed opt-out cards. At the other locations we posted fliers describing the study, inviting participation, and enrolled interested parents on interview days. We recruited providers for the key informant interviews using reputational case selection methods, targeting pediatricians from MCCHC, Chelsea-based dentists, hygienists, and staff from various early childhood program providers, (e.g. Women, Infant and Children (WIC), Early Head Start, preschool programs, etc). To determine the final sample size, we used “thematic saturation,” whereby parents were interviewed until no new themes emerge.

### Data collection

We conducted all interviews in English, in-person or by telephone. A female pediatrician, (II) conducted all semi-structured interviews with parent participants, while LD (a female dentist) conducted all provider interviews. We developed interview guides using our conceptual framework (Figure 1), and adapted questions from our previous work and published studies [26,27]. The guides were pilot tested and included a core list of open-ended questions and probes, but allowed for exploration of unanticipated lines of inquiry. Questions explored parents’ OH attitudes and beliefs (e.g. importance of baby teeth, knowledge of dental services/resources in Chelsea; age 1 year visit, timing of seeking dental care (preventive or emergency) and how this compares to medical care seeking; perceptions of barriers to PDC; and suggested strategies to address the problem. We pilot-tested the guide and obtained verbal consent from participants before each interview. All interviews were recorded and transcribed verbatim. Parent participants received a $20 gift card. Provider interview questions were designed to gain insight into perceptions about PDC, perceived facilitators, barriers and proposed solutions. This study was approved by the Massachusetts General Hospital Institutional Review Board.

### Data analysis

Data collection and analysis occurred simultaneously and analysis was comparative and thematic. Three researchers (LD, II, MG) reviewed the transcripts, independently coded and categorized the data. We triangulated and integrated results of parent interviews with data from key informant interviews and identified emerging themes. We used NVivo 10 data analysis software for coding and analyses. After integrating key themes from parent and provider interviews, we used them to refine our conceptual framework (Figure 1). These major themes are presented in the results.

## Results

Twenty-nine (29) parents completed the brief survey, while 24 parents completed semi-structured interviews. All respondents were mothers, majority of whom were between the ages 20–29 years (41%), Hispanic (48%), and had high-school or less education (55%). The mean age of children was 3.4 ± 1.2 years. Child and parent characteristics are displayed in Table 1. Of the parents surveyed, 66% of their children had ever had a dental visit, while only 31% had received the age 1 year dental visit. (Table 2) About a quarter (24%) of children reportedly had a dental problem (caries or history of trauma). Seventy-two percent (72%) of parents knew of a dentist to whom they could take their young child to, while 83% of parents knew of a dentist who accepts MassHealth.

Key informant interviews were conducted with 10 Chelsea-based providers, including 3 pediatricians, 2 dentists (one general dentist and one pediatric dentist), 1 dental hygienist and 4 early childhood program providers (e.g. Early Head Start, John Silber Early Learning Center, Woman Infant and Children (WIC) staff).

## Thematic Areas

### Theme 1: Barriers to receipt of preventive dental care

#### Financial Barriers

The most frequently cited barrier by parents (75%) to children’s receiving PDC was insurance related problems for children and adults. For children, lack of insurance was mostly an issue among children from undocumented immigrant families, who were unable to enroll in public or private insurance programs. All other children have some form of insurance that provides coverage for at least basic PDC. However, even among insured children, many parents are not aware of the dental benefits included as part of their health insurance coverage. Excessive out-of-pocket expenses was another real or perceived barrier mentioned by some parents, either from paying for services not covered by dental insurance, or from insurance co-pays. In addition, dental insurance for some families comprised only coverage for basic, preventive care and not for treatment. However, because some parents conflated preventive dental services with therapeutic dental care, they chose not to seek dental care for any reason, for fear of unaffordable out-of-pocket costs.

Of note, several respondents mentioned a lack of adult dental insurance coverage as an important barrier that influenced young children’s receipt of PDC in Chelsea. Many parents did not have insurance coverage for dental services for themselves, or were not aware if they did. Consequently, this barrier faced by parents translated into a perceived barrier to care for their children.

#### Structural barriers

Several structural barriers impeded children from receiving timely PDC. Although respondents mostly agreed that there was an adequate supply of dentists in Chelsea, some felt that the types of services available for children <2 years of age was limited. A few (5) parents noted that their pediatricians or dentists recommended that the child’s first dentist visit should occur at age 2 or 3 years, and so the child did not receive the age 1 year dental visit. Some dental providers in Chelsea were perceived as being uncomfortable or unwilling to see children <2 years, and this impacted receipt of the age 1 year visit. There was a general perception among some provider and parent respondents that the quality of care delivered at some for-profit dental practices in the community to be sub-par, with inadequate communication with parents about care delivered, and excessive emphasis on unnecessary dental procedures and treatments. This led some parents to travel out of the community to seek dental care elsewhere. Lastly, some elements of the organization of the health system in general, such as poor medical/ dental care-coordination and inadequate culturally appropriate care, were additional barriers that emerged.

#### Personal/Family-level barriers

Various personal/family-level factors were identified as resulting in foregone PDC among young children in Chelsea, e.g. parental lack of knowledge, low OH literacy and competing priorities. The low socio-economic status of many families meant that they often worked multiple jobs in order to make ends meet. This and other competing priorities resulted in their child’s PDC being forced down a list of other important tasks. About a quarter (25%) of parent respondents were not aware that children should have a dentist visit by age 1 year. Some preferred to wait until the child was old enough to sit in the dental chair, or had developed adequate expressive language skills. Of note, although 63% of parents felt that a child’s first dental visit should occur at age 1 year, only 31% of children had received the age 1 visit. A common personal-level barrier mentioned by non-parent respondents was the notion that parents perceived baby teeth as not important or indispensable. However, this attitude was not identified as an OH belief held by any of the parent-respondents.

Some parents reported that they or their child had a fear of the dentist, and this precluded them from receiving timely PDC. Other fears parents mentioned that resulted in foregone PDC included a fear that they would be unable to communicate their child’s needs adequately due to their limited English proficiency.

#### Community-level barriers

A few community-level factors were identified as potential influencers of children’s receipt of PDC. Some parents and providers noted that early childhood caries (ECC) was quite rampant among Chelsea children, many of whom had silver crowns as telltale signs of their poor OH status. This high prevalence of tooth decay shaped the community oral health (OH) environment. This OH environment may have resulted in tooth decay becoming an accepted community norm that could not be prevented. Further, because a large proportion of the community is comprised of immigrants from other parts of the world, some of the OH-related cultural norms and practices of their countries of origin also influenced the community’s OH environment. Some immigrant families reported that in their countries of origin, the norm was to seek care only for dental symptoms, and not for prevention. They continued this practice after immigrating to the US. Another construct that emerged within this theme was the issue of dental tourism; some families travel to their countries of origin to receive dental services, due to the high costs associated with dental care, or perceived low quality of care in the community.

### Theme 2: Facilitators of Receipt of Preventive Dental Care

#### Personal/Family-llvel facilitators

A majority of parent respondents indicated that prior dental experiences were an important driver of whether their children had received PDC. For example, parents who had experienced OH problems or dental pain in the past sought timely PDC on behalf of their children in order to protect them from experiencing similar problems. Other parents who did not receive routine dental care for themselves were less likely to take their child to the dentist. Some parents of children who had received the recommended age 1 year visit said they had done so because they learned about the importance of the age 1 year visit from an older child’s dental experience. Parents of children who had received the age 1 year visit reported the need to routinize PDC in their young child as a motivation for seeking care early. Changes in the child’s diet, as well as eruption of teeth were other motivating factors for the age 1 year visit.

Having a relationship with a family dentist was another personal-level factor that facilitated children’s PDC. Families that had a dentist that they trusted and had established a long-term relationship with over the years found it easier to take their child for PDC.

#### Health system facilitators

Dental services were perceived to be widely available in Chelsea. Most (68%) respondents felt that there were an adequate number of dental services and resources in the community, including for Medicaid enrollees. Examples of available dental services mentioned included private dentists, for-profit dental practices, school-based dentists, mobile vans, as well as dental services provided at Head- Start programs or other community locations. Many participants stated that most dentists accept MassHealth, and that dentists were located in easily accessible sites around the community. These findings were supported by survey results that indicated that majority of parents (87%) knew of a dentist to whom they could take their young child to, and that accepts MassHealth. The proximity to dental practices and ability to get there on foot or by public transportation was mentioned by several parents as facilitators of PDC.

Pediatricians were frequently cited as important facilitators of young children’s receipt of PDC. Some pediatric primary care clinics in the community provided preventive dental care (assessment, examination and fluoride varnish) as part of the well-child-visit, and referred parents to dentists in order for the child to establish a dental home. Parents saw pediatricians as reliable sources of OH information, and relied on them to determine at what age the child first dental visit should occurs. Pediatricians who had established relationships with dentists in the community reported that this made it easier for their young patients to receive timely PDC. Parents also relied on word-of-mouth dental referrals from family members and friends.

Some direct to consumer outreach activities implemented by various organizations also emerged as facilitators of PDC. For example, some parents received mailed reminders from MassHealth about the importance of the age 1 year dental visit, and therefore sought timely care. MassHealth also provides a list of dentists that see young children and accept MassHealth. Many dental providers advertise the fact that they see young children and accept MassHealth; some practices stationed their staff in pediatric offices to reach out to parents and inform them about their services. Fifty-percent (50%) of parent participants noted the child-centric resources (e.g. play areas) and services provided at some community dental practices as a facilitator of care; this created an impression of the practices’ competence in caring for children and made parents comfortable taking their children there.

#### Community/Policy-level Facilitators

Community/Policy-level factors that emerged as facilitators of young children’s receipt of PDC were identified by mostly providers and a few parents. They included OH policies in various early childhood programs, e.g. children enrolled in Head-Start are required to have routine dental exams. To ensure success of this policy, Head-Start programs staff (family advocates) support parents in various ways to help children receive timely PDC (e.g. provide transportation, establish linkages to dental homes). Additional OH related activities for Head-Start participants included: OH education classes for parents, and required daily tooth brushing for all head-start children. Families of children enrolled in WIC also received OH education as part of the nutrition education delivered routinely to participants. Dental practices and organizations also engaged the community in various OH community outreach activities that positively influenced the OH environment. Examples included OH fairs in the local libraries and other community locations, mobile dental vans, and media advertising.

### Theme 3: Proposed solutions

Various multi-level strategies were proffered as solutions to address identified barriers to receipt of PDC. Parent and provides respondents believed that parents should be key partners in efforts to improve young children’s utilization of PDC. Parent OH education was a recurring theme identified as critical to this process. Beyond educating parents on ECC and the importance of PDC, education was also perceived to be needed on how to navigate and access the myriad OH resources available in the community that parents be unaware of. Parents could also be educated about their family’s dental insurance benefits and the types of services it covers.

With regards to the health-care system, proposed solutions by parents and providers included: provide training/support for health care providers (e.g. train general dentists to treat young children, educate and support pediatricians to deliver PDC during well-child-visits), develop and disseminate an up-to-date list of area dentists who treat young children and accept MassHealth, provide appropriate and culturally-competent care (e.g. educate and serve families in languages they understand), implement sliding-scale payment systems, use multi-modal message delivery tools (e.g. multi-media, paper handouts, etc.), expand clinic hours, and enhance medical/dental integration. Proposed strategies to achieve better medical/dental integration included co-locating dental clinics within medical offices, and improving care coordination and communication between dental and medical care providers.

To address community-level factors, various local and policy level-strategies were proposed, including improved community engagement and outreach, particularly targeting and providing better support for populations that may be disconnected from the health care system, such as recent immigrants, refugees, and teenage mothers. Fostering improved collaboration among all providers that care for young children was a solution that emerged among early childhood program respondents. Strengthening partnerships between pediatricians, dentists, WIC, daycare and Head-Start staff could help provide a stronger support network for families so they do not fall through the cracks and are able to receive appropriate and timely preventive medical and dental care. At the policy-level, expansion of dental insurance coverage (for children and parents) was a recurring solution mentioned by most respondents.

## Discussion

We embarked on this study to gain an in-depth understanding of multi-level contextual factors that may influence receipt of preventive dental care among a vulnerable population of children living in Chelsea, MA. In our study sample, only 66% of children had ever had a dental visit, while 31% had received the age 1 year visit. We identified various personal/family, financial, structural, community and policy-level factors that were determined to be influential facilitators and barriers to young children’s receipt of PDC. An important financial barrier was dental insurance coverage-some children have no or limited coverage, which impacted their receipt of PDC. In addition, a lack of understanding of the dental benefits covered as part of their health insurance was another barrier among some insured families. It was interesting to note that adult dental insurance coverage was also perceived to play a role in children’s PDC. Because children and parents’ receipt of dental care is linked, this adult specific barrier was perceived in some way to impact children’s receipt of PDC. It is unclear through what specific mechanism adult dental insurance coverage could impact children’s receipt of PDC. We could not identify papers that have addressed this phenomenon in the literature. This finding should be further assessed in future studies, as it could shed light and inform policymakers on possible unintended consequences of eliminating adult dental coverage.

Some important structural barriers identified in our study included perceived low quality of preventive dental care or overemphasis on dental procedures with less prevention based care in some practices, as well as a lack of medical/dental coordination. Other studies have identified additional structural barriers, (e.g. transportation problems, limited clinic hours and difficulty making appointments) as reasons for foregone pediatric dental visits [28–30]. These kinds of barriers were not frequently mentioned as important factors in our study, perhaps as a result of the robust array of dental resources available in Chelsea.

The influence of dental tourism on children’ receipt of PDC was an unanticipated finding. Some families choose to seek care in their countries of origin, due to high costs associated with dental care in the US. This strategy could limit the family’s interaction with the US dental care system, and ultimately lead to delayed or foregone PDC in their children. Recently, there has been a growing body of literature documenting the increasing trend of dental tourism globally. Reasons often cited for traveling abroad for dental treatment include reduced costs, previous negative dental experiences, and easy access to quality dental care [31–33]. We could not identify any study that addressed the potential impact of dental tourism on children’s dental utilization.

With regards to facilitators of young children’s receipt of PDC, parents’ personal dental experience was one of the most frequently cited factors. This finding is consistent with other studies that demonstrated strong associations between parent and children’s dental utilization [34,35]. Among a low-income African-American population in Detroit, parents who had received PDC were five times as likely to seek care on behalf of their children, compared to parents without a history of a dental visit or who sought symptom-based dental care [36]. Our study validated findings from other studies on facilitators of young children’s dental utilization [37,38]. Among Mexican-American children living in California, various internal (e.g. parent’s desire to avoid future dental problems, pain or visible dental issues) and external (e.g. pediatrician recommendation, school-entry policy) prompts were identified as motivations for children’s first dental visit [38]. These and other external cues were often proposed by participants in our study as potential solutions to help address problem of foregone PDC.

Various OH policies implemented in early childhood programs were important facilitators of children’s receipt of PDC. Several systems were in place to help ensure the success of these policies, including strong Head Start/dental linkages and the use of family advocates to help families navigate the dental system. The success of these OH policies highlights opportunities to identify other policy levers that could be used as strategies to help ensure young children’s receipt of not just initial, but routine PDC. These policies could be expanded to impact other preschool-aged children not enrolled in Head Start, especially those not enrolled in organized pre-school programs, e.g. family daycares. Additional policies could also be implemented that encourage and support publicly insured families to receive timely and routine PDC. There is a possibility that such policies could place an undue burden on families that may have difficulty finding dentists willing to see their Medicaid-enrolled children. As such, implementing such a policy would require that certain systems like those implemented by Head-Start be put in place to support families and strengthen dental linkages. It would also be important to adopt systems that foster improved measurement, tracking, and surveillance in order to ensure delivery of high quality care that improves outcomes.

This study’s results should be interpreted with caution, given its limitations. We chose qualitative methodology to explore contextual factors that influence children’s receipt of PDC. This methodological approach allows researchers to gain a deeper understanding of individuals’ experiences, perspectives and interactions [39–40]. However, a limitation of this approach is that investigator bias could be introduced during data collection and analysis [40]. We tried to minimize some of these biases associated with qualitative research by obtaining data from multiple stakeholders with diverse perspectives, using a standardized interview-guide, employing rigorous, iterative qualitative data analyses procedures and triangulating results. Our findings may not be generalizable to other populations. The results represent the perceptions of thirty-four parents and providers in Chelsea, MA, and may not be representative of everyone in the community, or individuals from other communities. In spite of these limitations, this case study of the Chelsea community adds to the limited number of studies focusing on receipt of PDC among preschool-aged children. It adds to the literature by providing a multi-level, more diverse perspective that could be useful in validating or refuting results of other studies exploring influences of low-income children’s receipt of PDC. The community-generated solutions elicited in this study could be used to inform future quantitative and intervention studies designed to address the problem.

## Conclusion

Although there are a considerable number of dental resources in Chelsea, MA, various modifiable multi-level factors contribute to lack of access to these services by preschool-aged children. Opportunities exist to address modifiable factors through innovative solutions proposed by community stakeholders.

## Figures and Tables

**Table 1:** Child and Parent Characteristics.

*Child Characteristics*	n =29
Mean age (SD)	3.4 (1.2)
Insurance Status	
Private	14%
Public	62%
Uninsured	7%
Missing	17%
*Parent Characteristics*	
Mother	100%
Age Group	
≤ 19 years	14%
20–29 years	41%
30–39 years	31%
≥40 years	14%
Race	
Hispanic	48%
Black	31%
Other	21%
Parent Education	8%
≤High School	55%
Some College/2-year College	24%
≥College	21%
Single parent household	29%

**Table 2:** Child and Parent Dental Experience.

	n =29
Child ever had a dentist visit	66%
Child had a dentist visit at Age 1 year	31%
Child has an oral health problem	24%
Parent knows a dentist who sees young children	72%
Parent knows a dentist who accepts MassHealth	83%

**Table 3:** Themes and Sample Quotes.

Theme 1: Barriers to preventive dental services for preschool children in Chelsea, MA	
Construct	Sample Quotes
Structural Appropriateness Type of dental services Perceived quality of care Health system organization Provider characteristics Financial Insurance Reimbursement Out-of-pocket costs Personal/Family Socio-demographics Acculturation Oral health attitudes/literacy English proficiency Dental fear Immigration status Competing priorities Community Caries epidemic Cultural norms and practices Dental tourism	**Parent:** “When they had a little bit of decay, they used to just do bonding, just fill it in. Now, they have to cap the whole teeth; I wasn’t satisfied with that….maybe they get more out of insurance.” **Early childhood program provider:** “People don’t really get a lot of information… so I just think that the preventive side (of dentistry) is not very strong and that’s why you see so many kids (with cavities). **Pediatric Provider:** “These are baby teeth; they’re going to fall out. I won’t worry about it until their new teeth come in. So we’re up against some of those faulty notions about dental care.” **Dental provider:** “I think that among the Hispanic community, the prevalence of decay is so high that it’s looked at as just a normal part of growing up, and not as something that can be prevented.” **Pediatric Provider:** “I do feel like there’s not enough (dentists) comfortable with the younger age groups, who see our patients with Mass Health.” **Parent:** “Like in our culture, they cry, we give a lolly. And the truth I learned hear about doesn’t give too many candies to kids, and I thought well, I didn’t care about that. Now I know and I learned. When my kids are eating something, if its candy, I say go brush your teeth after. But I think we need more education about…” **Parent:** “Insurance (is a problem). Yeah, because sometimes we don’t know where to go to apply or…some people help us to find the right information…And sometimes we try and somebody say no…Well what can we do if nobody helps me?” **Interviewer:** “At what age do you think a child should have their first dental visit?” Parent: “2 years because they are, they have their first teeth. And they need to take care of them. I haven’t taken my daughter yet, but I see her teeth…I mean asking them to brush their teeth every time before they go to bed at night. And I think her teeth are alright. They are clean. They are no black spots on them.” **Pediatric Provider:** “It’s hard, a lot of my patients are cared for by other family members, who may be even more recent arrivals to the country, and so they may not have a lot of information or familiarity with dental prevention for little kids. So I don’t know how to reach all of those parents that don’t interact with any of the services. I think WIC would be good, because WIC sees everyone. And I don’t know if they’re going to do something like that, I’m not sure if they do or they don’t.” **Early childhood program provider:** “…first of all that parents don’t have any coverage for themselves. And the adults don’t get any dental…they don’t have financial access to dental services. And without that being kind of integrated into the family for all members, then I think it’s a little harder to get it integrated for the children.ldvn16 **Parent:** “Well, my situation when I was staying with my father. My father didn’t understand English well. So, all he was saying was yes, yes, yes, just for him to like-- because he knows I was in pain. So, they pulled out my two front teeth without his-- well, nobody was there to explain it to him. So, when he noticed that they were pulling out my teeth he was really frustrated, really angry at them.” **Interviewer:** “At what age do you think a child should have their first dental visit?” **Parent:** “Age two. Early on at one if they’re not speaking they might act out. Then you might have to hold them down and then they might resent that you’re holding them at one-year-old to look in their mouth, because they’re very agile. But you can explain to them that they are going to the dentist. And then at that age most of the teeth are coming in already. That’s why I pick age two.”
**Theme 2: Facilitators of preventive dental services for preschool children in Chelsea, MA**	
**Construct**	**Sample Quotes**
Personal/Family Prior dental experience Family dentist Health System Availability Accessibility Continuity of care Child-centric services Efficacy of treatment Pediatrician referrals Medical/Dental Integration Policy/Community Environment Outreach and engagement Early childhood programs Oral health education Oral health policies Schools	**Parent:** “I am almost 40 years, and when I go to the dentist now, I cry when they have to take away my teeth. I really feel bad! I do not want my child to go through the same thing I did.” **Parent:** “…there are so many dentists in Chelsea. Chelsea is not very big…. I like that we have choices. There are many choices. If you don’t like one, then there are other dentists available and you don’t have to go too far.” **Dental provider:** “We have a variety of services; when we talk to our counterparts throughout the state we always talk about how fortunate we are in Chelsea to have really good access to dental care.” **Parent:** “…her pediatrician gave me a list of dentists around Chelsea and close to me; …there were some (dentist) representatives down on the first floor (of the pediatric office) and they were giving out papers and actually making appointments the same day for them to check on kids.” **Pediatrician:** “I have fostered a relationship with the dentists in the area, so I know them, and I can call their office and beg for my patients to be seen sooner.” **Parent:** “I think that (the dentists) do a pretty good job of letting it be known that they take any kind of insurance, so people come.” **Parent:** I wanted him to get into the routine because I’ve seen a lot of other kids that they are so afraid of the dentist, and I wanted him to see that there is nothing to be afraid of. The younger he saw them the better it is. **Parent:** “Well, I’m a person it’s important for me to go to the dentist. If the baby has enough teeth and they’re eating different types of food with sugar, which is candy, just as soon as possible just for them to prevent having cavities in the early stage.” **Interviewer:** “At what age do you think a child should have their first dental visit?” **Parent:** “I year old. Before, I did not have any idea – for my first child I lived in NewYork and they sent us a card. But I could not read the paper, and so I did not know. Here in Chelsea, when MassHealth sent the card (to tell me to take my child to the dentist), I went to the doctor, I asked when should my child go to the dentist, - she said when they are 6 months they should go to the dentist, just to see how the teeth are growing. So I think it is better when the child is 6monhts or 1 year.”

## Abbreviations

PDC: Preventive Dental Care
OH: Oral Health
ECC: Early Childhood Caries
